# Risk factors and comorbidities for invasive pneumococcal disease in Western Australian Aboriginal and non-Aboriginal people

**DOI:** 10.15172/pneu.2014.4/463

**Published:** 2014-12-01

**Authors:** Faye J. Lim, Deborah Lehmann, Aoiffe McLoughlin, Catherine Harrison, Judith Willis, Carolien Giele, Anthony D. Keil, Hannah C. Moore

**Affiliations:** 14 Telethon Kids Institute, The University of Western Australia, PO Box 855, 6872 Perth, Western Australia Australia; 24 Western Australian Department of Health, Communicable Disease Control Directorate, Perth, Western Australia Australia; 34 Department of Microbiology, PathWest Laboratory Medicine Western Australia, Princess Margaret Hospital for Children, Perth, Western Australia Australia

**Keywords:** Invasive pneumococcal disease, risk factors, comorbidities, pneumonia, immunization

## Abstract

Australian Aboriginal people have among the highest rates of invasive pneumococcal disease (IPD) worldwide. We investigated clinical diagnosis, risk factors, comorbidities and vaccine coverage in Aboriginal and non-Aboriginal IPD cases. Using enhanced surveillance, we identified IPD cases in Western Australia, Australia, between 1997 and 2007. We calculated the proportion with risk factors and comorbidities in children (<5 years) and adults (=15 years), as well as adults living in metropolitan and non-metropolitan regions. We then calculated the proportion of cases eligible for vaccination who were vaccinated before contracting IPD. Of the 1,792 IPD cases that were reported, 355 (20%) were Aboriginal and 1,155 (65%) were adults. Pneumonia was the most common diagnosis (61% of non-Aboriginal and 49% of Aboriginal adult IPD cases in 2001–2007). Congenital abnormality was the most frequent comorbidity in non-Aboriginal children (11%). In Aboriginal children, preterm delivery was most common (14%). Ninety-one percent of non-Aboriginal and 96% of Aboriginal adults had one or more risk factors or comorbidities. In non-Aboriginal adults, cardiovascular disease (34%) was the predominant comorbidity whilst excessive alcohol use (66%) was the most commonly reported risk factor in Aboriginal adults. In adults, comorbidities were more frequently reported among those in metropolitan regions than those in non-metropolitan regions. Vaccination status was unknown for 637 of 1,082 cases post-July 2001. Forty-one percent of non-Aboriginal and 60% of Aboriginal children were eligible for vaccination but were not vaccinated. Among adults with risk factors who were eligible for vaccination and with known vaccination status, 75% Aboriginal and 94% non-Aboriginal were not vaccinated. An all-of-life immunisation register is needed to evaluate vaccine coverage and effectiveness in preventing IPD in adults.

## 1. Introduction

Invasive pneumococcal disease (IPD) refers to diseases resulting from *Streptococcus pneumoniae* infecting a normally sterile site, such as the blood stream and cerebrospinal fluid [1]. Those at highest risk of IPD are children aged less than 2 years, adults aged over 65 years, Indigenous populations in industrialised countries, those living in third world settings, persons with underlying chronic conditions including cancer, diabetes, cardiac or renal disease, and those consuming excessive alcohol and tobacco [2, 3].

IPD became notifiable in Australia in 2001 [1]. In 2005–2010, on average 1,560 cases of IPD were reported annually in Australia, with notification rates between 7.0 and 8.3 per 100,000 [4]. Between 2003 and 2006, the overall rate of IPD in Australia was over 4 times higher in the Aboriginal and Torres Strait Islander population (herein referred to as Aboriginal) than in the non-Aboriginal population [5]. The difference was much greater in young adults aged 25–49 years, with IPD rates more than 11 times higher in young Aboriginal adults than in their non-Aboriginal peers [5].

Clinical manifestations of IPD include pneumonia, septicaemia, bacteraemia and meningitis [5, 6]. The most common clinical manifestation of IPD in adults is pneumonia; estimated to account for up to 90% of adults with IPD [6, 7]. By contrast, children with IPD most commonly present as having bacteraemia [6].

Vaccines targeting *S. pneumoniae* have been available in Australia since the 1980s for those at increased risk of developing IPD. The 7-valent pneumococcal conjugate vaccine (7vPCV) was introduced in 2001 for Aboriginal children aged less than 2 years in a 3-dose schedule at 2, 4 and 6 months of age, and for children with predisposing medical conditions such as immune deficiency and chronic lung disease [8]. In 2005, the program was expanded to include non-Aboriginal children [9]. A funded immunisation program for the 23-valent pneumococcal polysaccharide vaccine (23vPPV) was introduced in 1999 for Aboriginal adults aged 50 years or more and Aboriginal adults aged 15–49 years with known risk factors [10], and from 2005 onwards, for non-Aboriginal adults aged 65 years or more [9]. Between 2001 and 2011, 23vPPV was also offered to those at high risk of IPD, including Aboriginal children at age 18 months as a booster dose, and adults who smoked or had chronic diseases [8, 9].

In Western Australia (WA), Australia, enhanced surveillance of IPD began in 1996. For the period 1997–2007, the average annual IPD incidence rate in WA was 8.4 per 100,000 [11]. Incidence of IPD was 6.7 times higher in the Aboriginal population than in the non-Aboriginal population, with the greatest disparity in adults aged 30–49 years in the period 2005–2007 [11]. Since 2000, there has been a reduction in the rates of IPD caused by serotypes included in 7vPCV in all children aged less than 2 years [11]. At the same time, there was an emergence of non-7vPCV serotypes, particularly 19A [12]. Using our enhanced surveillance data from 1997 to 2007, we present a descriptive analysis of the clinical diagnosis of IPD cases in WA and the changes over time, their documented risk factors and comorbidities by age, Aboriginal status and geographical regions, as well as their vaccination status before contracting IPD.

## 2. Methods

### 2.1 Setting and Study Population

**Figure 1:**
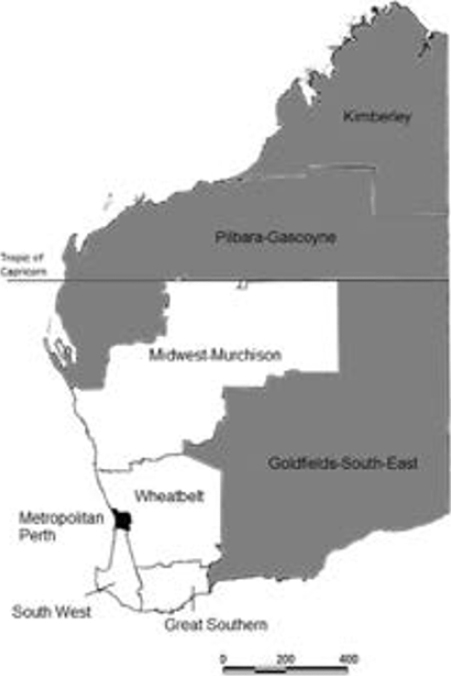
Map of Western Australia showing the eight geographical regions. Metropolitan regions (Perth) are shaded in black (■), rural regions (South West, Great Southern, Wheatbelt and Midwest-Murchison) are in white (□), and remote regions (Kimberley, Pilbara-Gascoyne and Goldfields-South-East) are shaded grey ().

The Department of Health, WA, has classified WA into 8 geographical regions: metropolitan Perth; the Great Southern, South West, Wheatbelt and Midwest-Murchison regions which form rural WA; and the Kimberley, Pilbara-Gascoyne and Goldfields-South East regions which form remote WA (Figure 1). The Aboriginal population accounts for 3.8% of the state’s population [13]. Over the study period (1997–2007), 64% of the Aboriginal population and 22% of the non-Aboriginal population were living in rural or remote regions of WA [13].

### 2.2 Data collection

Enhanced surveillance of IPD in WA was conducted through the Vaccine Impact Surveillance Network (VISN) from 1997 to 2007 [11, 14]. IPD cases occurring in public and private hospitals between 1997 and 2007 were identified through surveillance of private and public laboratories for isolation of *S. pneumoniae* from normally sterile sites. When IPD became notifiable in 2001, cases were also identified from reports issued by the Department of Health of WA.

Data were collected through a review of medical records and recorded on a standard data collection sheet. We collected data on clinical diagnosis; known risk factors such as excessive alcohol use; smoking; chemotherapy or steroid use; immunocompromised state such as HIV and malignancies, and chronic diseases such as diabetes, respiratory and cardiovascular disease. Final clinical diagnosis was recorded as either pneumonia, meningitis, otitis media, bacteraemia, septicaemia, other, or unknown. We defined risk factors as potentially modifiable behaviours or conditions (e.g. smoking or steroid use) or known risk factors for infectious diseases (e.g. preterm delivery) while all other conditions were classified as comorbidities. Preterm delivery was defined as less than 37 weeks gestation. Excessive alcohol use was defined as alcohol abuse as recorded in medical notes. Chronic respiratory disease was defined as the presence of one of more of the following: chronic obstructive airways, asthma or other pulmonary conditions, including bronchiectasis and asbestosis.

The data collection sheet was modified in 2001 to capture more detailed information in all areas and to systematically collect vaccination data. Data collected from 2001 onwards, included date and type of pneumococcal vaccination and additional comorbidities such as asplenia and congenital abnormalities. Immunisation data were sourced from medical records or the Australian Childhood Immunisation Register [15].

### 2.3 Statistical Analysis

IPD cases were grouped by age: children aged less than 5 years, children aged 5–14 years and adults aged 15 years or more. A hierarchical diagnosis algorithm was developed for cases with multiple diagnoses and based on the following order of disease severity: meningitis, septicaemia, pneumonia, bacteraemia and other diagnosis (e.g. otitis media or other non-specific clinical foci). The proportion of cases with each clinical diagnosis and the exact 95% confidence interval were then calculated for the two time periods 1997–2000 and 2001–2007. For analyses of common risk factors and comorbidities in adults, all IPD cases in Aboriginal adults aged 50 years or more between 1997 and 2007 were analysed together due to small numbers. The proportions of Aboriginal and non-Aboriginal cases with each risk factor and comorbidity were calculated in each age group with exact 95% confidence intervals for the period 1997–2007. We conducted chi-squared tests comparing proportions between groups. Proportions of cases with any risk factor or comorbidity were only calculated on cases with complete data on all the risk factors and comorbidities listed. Geographical region was assigned using individual’s postcode at the time of contracting IPD.

Vaccination status was only analysed in cases occurring after July 2001 when data on vaccination status were routinely collected. As 7vPCV was only funded for non-Aboriginal children from 2005 [9], children aged less than 5 years were separated into 3 groups: Aboriginal (2001–2007), non-Aboriginal with risk factors (2001–2004) and non-Aboriginal (2005–2007) to represent the groups eligible for vaccination. Risk factors predisposing children to IPD for which 7vPCV is recommended include immunodeficiency, chemotherapy or steroid use, asplenia, renal failure, Down syndrome and preterm birth [8].

Adults were separated into 4 groups for analysis: non-Aboriginal aged 15–64 years with risk factors (2001–2007), non-Aboriginal aged 65 years or more (2005–2007), Aboriginal aged 15–49 years with risk factors (2001–2007) and Aboriginal aged 50 years or more (2001–2007). Risk factors predisposing adults to IPD for which 23vPPV is recommended include asplenia, immunodeficiency, chronic cardiac, renal or respiratory disease and smoking [8]. Due to small numbers, cases were coded as vaccinated if they received 1 or more doses of either 7vPCV or 23vPPV before contracting IPD. Analyses were conducted in IBM SPSS Statistics for Windows version 22 software [16] and EpiBasic version 1.0 [17].

### 2.4 Ethics

Ethical approvals to conduct the study were obtained from the Department of Health Western Australia Human Research Ethics Committee (Approval reference number: 2011/43) and the Western Australian Aboriginal Health Information and Ethics Committee (Approval reference number: 354-07/11).

## 3. Results

Between 1997 and 2007, there were a total of 1,792 cases of IPD reported in WA (990 male sex, 795 female sex, 7 unknown gender) of which 188 (10.5%) were known to have died (154 non-Aboriginal, 32 Aboriginal, 2 Aboriginal status unknown). There were 355 (19.8%) Aboriginal cases, and 24 cases where Aboriginal status was unknown. Overall, there were 532 (29.7%) cases aged less than 5 years, 103 (5.7%) cases aged 5–14 years, 1,155 (64.5%) adult cases and 2 cases of unknown age. Excluding cases of unknown age and Aboriginal status, 1,766 IPD cases were available for analysis. The majority of non-Aboriginal adult IPD cases were aged 65 years or more (46.0%), whereas the majority of Aboriginal adult IPD cases were aged 30–49 years (58.9%).

Of the 1,766 IPD cases, clinical diagnosis was unknown for 19 (14 non-Aboriginal, 5 Aboriginal). Overall, pneumonia was diagnosed in 163 (31.2%) children aged less than 5 years, 42 (41.6%) children aged 5–14 years and 726 (63.5%) adults aged 15 years and over. With the exception of non-Aboriginal children aged less than 5 years, pneumonia was the most common clinical diagnosis among children and adults with IPD in the periods before (1997–2000) and after (2001–2007) statutory notification and introduction of 7vPCV (Table 1). In non-Aboriginal children aged less than 5 years, bacteraemia was the predominant clinical diagnosis (38.5% of cases in 2001–2007) (Table 1). The proportion of adults diagnosed with pneumonia decreased significantly over the two time periods, although the decline was greater among Aboriginal adults (76.5% to 49.2%; *p* < 0.001) than in non-Aboriginal adults (75.8% to 60.7%; *p* < 0.001) (Table 1).

**Table 1:** Clinical diagnosis among Western Australian non-Aboriginal and Aboriginal population with invasive pneumococcal disease, 1997–2007

Group	Non-Aboriginal						Aboriginal					
	1997–2000			2001–2007			1997–2000			2001–2007		
	*n*	%	(95% CI)	*n*	%	(95% CI)	*n*	%	(95% CI)	*n*	%	(95% CI)
Clinical diagnosis												
Children (<5 years)												
Meningitis	17	9.6	(5.7–14.9)	32	12.3	(8.6–16.9)	6	16.2	(6.2–32.0)	6	12.8	(4.8–25.7)
Septicaemia	30	16.9	(11.7–23.2)	59	22.7	(17.8–28.3)	5	13.5	(4.5–28.8)	8	17.0	(7.7–30.8)
Pneumonia	57	32.0	(25.2–39.4)	66	25.4	(20.2–31.1)	16	43.2	(27.1–60.5)	24	51.1	(36.1–65.9)
Bacteraemia	59	33.1	(26.3–40.6)	100	38.5	(32.5–44.7)	7	18.9	(8.0–35.2)	9	19.1	(9.2–33.3)
Other^a^	13	7.3	(4.0–12.2)	2	0.8	(0.1–2.8)	2	5.4	(0.7–18.2)	0	0	(0.0–7.6)
Unknown	2	1.1	(0.1–4.0)	1	0.4	(0.0–2.1)	1	2.7	(0.1–14.2)	0	0	(0.0–7.6)
Adults (≥15 years)												
Meningitis	24	7.9	(5.2–11.6)	31	5.1	(3.5–7.2)	3	5.9	(1.2–16.2)	13	7.0	(3.8–11.7)
Septicaemia	24	7.9	(5.2–11.6)	157	26.0	(22.5–29.6)	3	5.9	(1.2–16.2)	64	34.6	(27.8–41.9)
Pneumonia	229	75.8	(70.6–80.6)	367	60.7	(56.6–64.6)	39	76.5	(62.5–87.2)	91	49.2	(41.8–56.6)
Bacteraemia	13	4.3	(2.3–7.3)	43	7.1	(5.2–9.5)	3	5.9	(1.2–16.2)	14	7.6	(4.2–12.4)
Other^a^	5	1.7	(0.5–3.8)	5	0.8	(0.3–1.9)	3	5.9	(1.2–16.2)	0	0	(0.0–2.0)
Unknown	7	2.3	(0.9–4.7)	2	0.3	(0.0–1.2)	0	0	(0.0–7.0)	3	1.6	(0.3–4.7)

Note: Some percentages may not total to 100 due to rounding.

CI, confidence interval.

^a^Includes otitis media and other non-specific clinical foci such as sinusitis and glomerulonephritis.

A third (33.7%) of non-Aboriginal and 38.9% of Aboriginal children aged less than 5 years had one or more risk factors or comorbidities at the time of contracting IPD (Table 2). Congenital abnormality was the most commonly reported comorbidity in non-Aboriginal children aged less than 5 years with IPD (11.2%). Among similarly aged Aboriginal children with IPD, preterm delivery was the most prevalent risk factor (13.9%) (Table 2). By contrast, 49.0% of non-Aboriginal and 40.0% of Aboriginal children aged 5–14 years had at least one risk factor or comorbidity; chronic respiratory disease was the most frequent comorbidity in Aboriginal children in this age group (8.8%). Chemotherapy or steroid use was reported in 14.9% of non-Aboriginal children aged 5–14 years (data not shown).

**Table 2:** Frequency of risk factors and comorbidities for invasive pneumococcal disease (with exact 95% confidence intervals) in Western Australian Aboriginal and non-Aboriginal children <5 years, 1997–2007

Group	Non-Aboriginal (*n* = 438)			Aboriginal (*n* = 84)		
	*n*	%	(95% CI)	*n*	%	(95% CI)
Risk factors						
Chemotherapy or steroid use	14	3.2	(1.8–5.3)	4	4.8	(1.3–11.7)
Preterm delivery	39	9.6	(6.9–12.9)	11	13.9	(7.2–23.6)
Any risk factor^a^	51	12.5	(9.5–16.1)	13	16.5	(9.1–26.5)
Comorbidities						
Congenital abnormality	29	11.2	(7.6–15.7)	2	4.3	(0.5–14.8)
Chronic respiratory disease	25	5.7	(3.7–8.3)	7	8.3	(3.4–16.4)
Cardiovascular disease	11	2.5	(1.3–4.5)	3	3.6	(0.7–10.1)
Disease of nervous system	10	3.9	(1.9–7.0)	2	4.4	(0.5–15.2)
Malignancy	8	1.9	(0.8–3.7)	1	1.3	(0.0–6.9)
Disease of digestive system^b^	5	1.9	(0.6–4.5)	1	2.2	(0.1–11.8)
Asplenia	2	0.8	(0.1–2.8)	0	0	(0.0–7.7)
Chronic renal failure	1	0.2	(0.0–1.3)	1	1.3	(0.0–6.9)
Hypertension	1	0.4	(0.0–2.2)	1	2.3	(0.1–12.0)
Anycomorbidities^a^	61	23.2	(18.3–28.8)	13	26.5	(15.0–41.1)
Any risk factors or comorbidities^a^	92	33.7	(28.1–39.6)	21	38.9	(25.9–53.1)

Note: Denominators for risk factors and comorbidities vary due to missing data. Aboriginal status was unknown in 10 cases.

CI, confidence Interval.

^a^Proportions with any risk factors or comorbidities were calculated from those with complete data on all listed risk factors and comorbidities.

^b^Gastro-oesophageal reflux disease (GORD) (*n* = 1), GORD and recurrent pancreatitis (*n* = 1), unspecified (*n* = 4).

In contrast to children aged less than 5 years, Aboriginal and non-Aboriginal adults had a significantly higher proportion of one or more risk factors or comorbidities, 95.9% (*p* < 0.001) and 90.9% (*p* < 0.001), respectively (Table 3). Excessive alcohol use was almost 5 times higher while smoking was twice as high in Aboriginal adults with IPD than in non-Aboriginal adults (both *p* < 0.001) (Table 3). Conversely, malignancy was almost 13 times higher and chemotherapy or steroid use was 3 times higher in non-Aboriginal adults with IPD than in Aboriginal adults (both *p* < 0.001) (Table 3). The proportion of non-Aboriginal adults who were ex-smokers was double that of Aboriginal adults (*p* < 0.001) (Table 3).

**Table 3:** Frequency of risk factors and comorbidities for invasive pneumococcal disease (with exact 95% confidence intervals) in Western Australian Aboriginal and non-Aboriginal adults aged 15 years or more, 1997–2007

Group	Non-Aboriginal (*n* = 907)			Aboriginal (*n* = 236)		
	*n*	%	(95% CI)	*n*	%	(95% CI)
Risk factors						
Smoker	240	28.2	(25.2–31.4)	116	60.4	(53.1–67.4)
Ex-smoker	115	19.6	(16.5–23.0)	12	6.9	(3.6–11.8)
Chemotherapy or steroid use	164	18.1	(15.6–20.8)	14	5.9	(3.3–9.8)
Excessive alcohol use	115	13.5	(11.3–16.0)	137	66.2	(59.3–72.6)
Any risk factor^a^	485	53.5	(50.2–56.8)	183	77.5	(71.7–82.7)
Comorbidities						
Cardiovascular disease	309	34.1	(31.0–37.3)	75	31.8	(25.9–38.1)
Chronic respiratory disease	275	30.3	(27.3–33.4)	56	23.7	(18.5–29.7)
Hypertension	159	26.9	(23.4–30.7)	43	24.4	(18.3–31.5)
Disease of digestive system^b^	150	25.2	(21.8–28.9)	59	33.0	(26.1–40.4)
Malignancy	200	23.2	(20.4–26.2)	4	1.8	(0.5–4.6)
Diabetes	130	14.9	(12.6–17.5)	97	42.9	(36.4–49.7)
Mental health conditions	79	13.2	(10.6–16.1)	12	6.7	(3.5–11.4)
Disease of nervous system	74	12.4	(9.9–15.4)	23	12.9	(8.4–18.8)
Chronic renal failure	69	8.0	(6.3–10.0)	46	21.2	(16.0–27.2)
Chronic liver failure/cirrhosis	49	5.7	(4.3–7.5)	45	21.0	(15.8–27.1)
Congenital abnormality	17	2.8	(1.7–4.5)	6	3.3	(1.2–7.0)
Asplenia	14	2.3	(1.3–3.9)	9	4.9	(2.3–9.1)
Any comorbidities^a^	626	83.5	(80.6–86.1)	163	81.9	(75.9–87.0)
Any risk factors or comorbidities^a^	722	90.9	(88.7–92.8)	210	95.9	(92.3–98.1)

Note: Denominators for risk factors and comorbidities vary due to missing data. Aboriginal status was unknown in 12 cases.

CI, confidence interval.

^a^Proportions with any risk factors or comorbidities were calculated from those with complete data on all listed risk factors and comorbidities.

^b^Includes gastro-oesophageal reflux disease (*n* = 18), pancreatitis (*n* = 12), oesophagitis (*n* = 11) and diverticular disease (*n* = 9).

**Figure 2:**
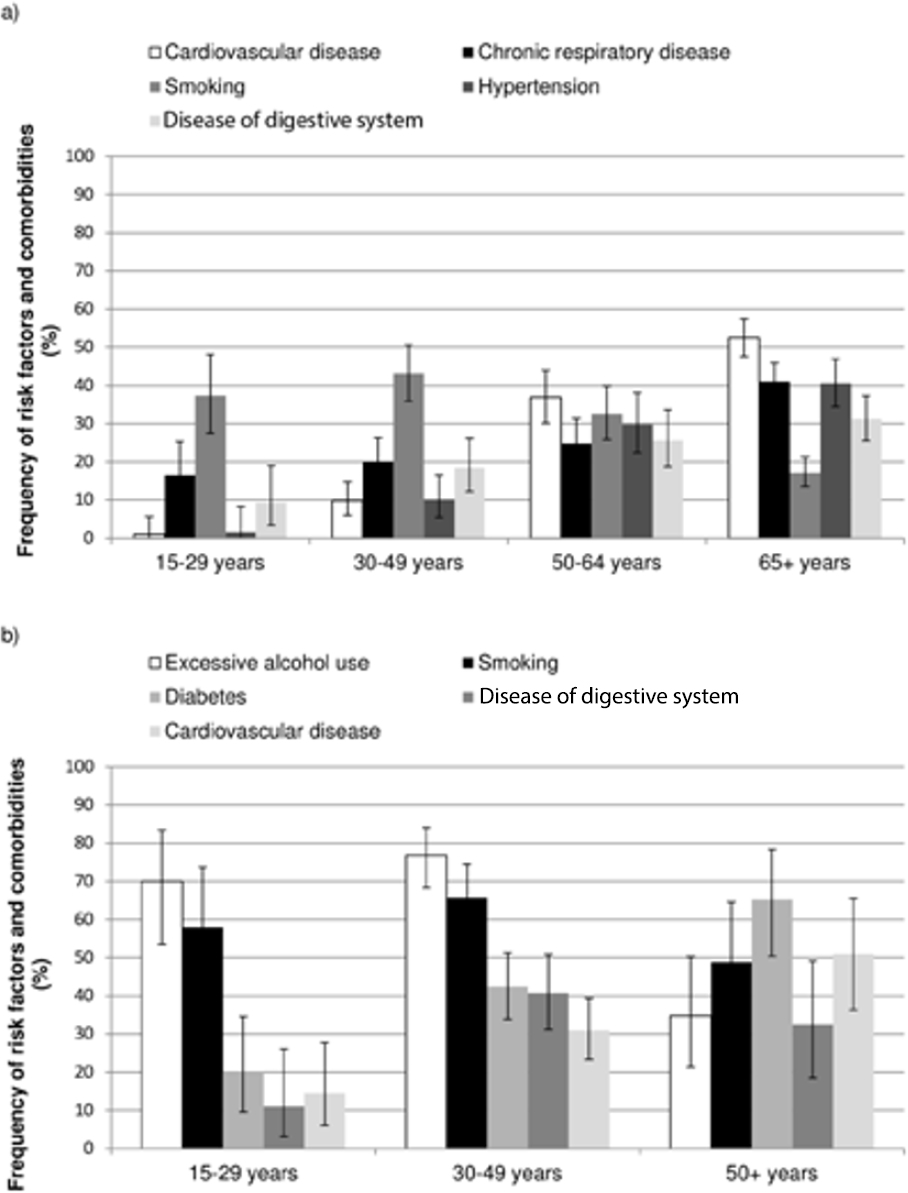
Frequency of common risk factors and comorbidities for invasive pneumococcal disease in a) non-Aboriginal and b) Aboriginal adults with exact 95% confidence intervals. Note: Aboriginal adults aged 50–64 years and 65+ years were grouped together due to small numbers

Smoking was the most common risk factor in non-Aboriginal adults aged less than 50 years, with twice the proportion of adults smoking as having any other risk factor or comorbidity in these age groups (Figure 2a). Among non-Aboriginal adults aged 65 years and over, cardiovascular disease was the most frequent comorbidity (Figure 2a). For Aboriginal adults, excessive alcohol use and smoking were the most common risk factors in those aged less than 50 years while in those aged 50 years or more, diabetes was the most prevalent comorbidity (Figure 2b). Aboriginal and non-Aboriginal children and adults presenting with pneumonia had a similar profile of risk factors and comorbidities to that of the overall group of children and adults with IPD.

Thirty-four IPD cases were either missing postcode of residence or did not live in WA: 33 non-Aboriginal cases and 1 Aboriginal case, of which, 3 were children and 31 were adults. The majority of non-Aboriginal (83.4%) and 32.3% of Aboriginal adults with IPD were living in the Perth metropolitan region. One fifth (21.3%) of Aboriginal adults with IPD were living in the Kimberley region (data not shown). The overall proportions of Aboriginal and non-Aboriginal adults with any reported comorbidities were higher among those living in the metropolitan region than among those living in rural or remote regions (Table 4). However, hypertension, diabetes and chronic renal failure were more common in Aboriginal adults living in remote regions than in their counterparts in metropolitan regions (Table 4).

**Table 4:** Number and percentage of Western Australian Aboriginal and non-Aboriginal adults aged 15 years or more with risk factors and comorbidities for invasive pneumococcal disease by geographical region, 1997–2007

Group	Metropolitan^c^ (*n* = 808)				Rural^d^ (*n* = 118)				Remote^e^ (*n* = 186)			
	Non-Aboriginal		Aboriginal		Non-Aboriginal		Aboriginal		Non-Aboriginal		Aboriginal	
	*n*	(%)	*n*	(%)	*n*	(%)	*n*	(%)	*n*	(%)	*n*	(%)
Risk factors												
Smoker	188	(27.1)	50	(70.4)	29	(33.0)	13	(61.9)	16	(38.1)	52	(52.5)
Ex-smoker	89	(19.5)	5	(9.8)	15	(20.0)	2	(12.5)	4	(11.1)	5	(4.8)
Chemotherapy or steroid use	132	(18.0)	8	(10.5)	21	(22.1)	0	(0)	7	(14.0)	6	(4.4)
Excessive alcohol use	87	(12.6)	48	(67.6)	18	(20.0)	17	(73.9)	9	(20.9)	72	(64.3)
Any risk factor^a^	381	(52.0)	69	(90.8)	61	(64.2)	19	(82.6)	26	(52.0)	94	(69.1)
Comorbidities												
Cardiovascular disease	256	(35.0)	24	(31.6)	31	(32.6)	6	(26.1)	15	(30.0)	44	(32.4)
Chronic respiratory disease	222	(30.3)	22	(28.9)	27	(28.4)	4	(17.4)	15	(30.0)	30	(22.1)
Hypertension	132	(28.8)	9	(17.0)	15	(19.2)	5	(29.4)	8	(22.9)	28	(26.7)
Disease of digestive system^b^	120	(25.9)	20	(37.7)	18	(23.4)	8	(47.1)	7	(19.4)	31	(28.7)
Malignancy	158	(22.8)	2	(3.0)	27	(29.3)	0	(0)	10	(20.8)	2	(1.6)
Diabetes	113	(16.2)	27	(37.5)	8	(8.5)	7	(31.8)	5	(10.0)	63	(48.1)
Mental health condition	65	(13.9)	5	(9.4)	11	(14.1)	2	(11.8)	0	(0)	4	(3.7)
Disease of nervous system	62	(13.4)	8	(15.1)	8	(10.3)	4	(23.5)	1	(2.8)	10	(9.3)
Chronic renal failure	57	(8.3)	13	(18.6)	5	(5.4)	2	(9.1)	5	(10.0)	31	(25.0)
Chronic liver failure/cirrhosis	43	(6.3)	20	(28.2)	4	(4.3)	6	(27.3)	1	(2.0)	19	(15.8)
Congenital abnormality	13	(2.8)	4	(7.5)	3	(3.8)	0	(0.0)	1	(2.7)	2	(1.8)
Asplenia	11	(2.4)	2	(3.8)	2	(2.6)	2	(11.1)	0	(0.0)	5	(4.8)
Any comorbidities^a^	508	(85.1)	59	(88.1)	66	(75.9)	14	(77.8)	30	(73.2)	89	(78.8)
Any risk factors or comorbidities^a^	581	(91.6)	73	(100.0)	77	(87.5)	20	(95.2)	38	(86.4)	116	(93.5)

Note: Denominators for risk factors and comorbidities vary due to missing data. Geographical region was unknown in 31 adult cases

^a^Proportions with any risk factors or comorbidities were calculated from those with complete data on all listed risk factors and comorbidities

^b^Includes gastro-oesophageal reflux disease (*n* = 18), pancreatitis (*n* = 12), oesophagitis (*n* = 11) and diverticular disease (*n* = 9)

^c^Perth region

^d^South West, Great Southern, Wheatbelt and Midwest-Murchison regions

^e^Kimberley, Pilbara-Gascoyne and Goldfields-South-East regions

There were 1,082 IPD cases that occurred after July 2001 which were available for analysis of vaccination status. Vaccination status was unknown in 637 cases (58.9%). Overall, a higher proportion of non-Aboriginal children (2005–2007) than Aboriginal children (2001–2007) received at least one pneumococcal vaccination before contracting IPD (58.8% compared to 40.5%; *p* = 0.07). Of the 141 Aboriginal adults aged 15–49 years with known vaccination status, 126 (89.4%) cases had risk factors predisposing them to IPD and were therefore eligible for vaccination with 23vPPV [8]. Of those with risk factors, 64 had vaccination status reported, 25.0% of whom were vaccinated (Table 5). Similarly, 246 (73.7%) of 334 non-Aboriginal adults aged 15–64 years had risk factors predisposing them to IPD [8]; 50 had vaccination status reported, of whom, 6.0% were vaccinated (Table 5). Of the adults eligible for vaccination due to the presence of risk factors, vaccination coverage was over four times higher in Aboriginal adults aged 15–49 years (25.0%) than in non-Aboriginal adults aged 15–64 years (6.0%; *p* = 0.01) (Table 5).

**Table 5:** Vaccination status of Western Australian Aboriginal and non-Aboriginal children and adults with invasive pneumococcal disease who were eligible for pneumococcal vaccination, 2001–2007

Group	Year group	Status unknown		Status known			
				Vaccinated		Not vaccinated	
		*n*	(% of total group)	*n*	(%)^a^	*n*	(%)^a^
Children (<5 years)							
Non-Aboriginal with risk factors	2001–2004	8	(25.0)	0	(0)	24	(100.0)
Non-Aboriginal^b^	2005–2007	3	(5.6)	30	(58.8)	21	(41.2)
Aboriginal	2001–2007	1	(2.3)	17	(40.5)	25	(59.5)
Adults (≥15 years)							
Non-Aboriginal (≥65 years)^b^	2005–2007	216	(85.7)	8	(22.9)	27	(77.1)
Non-Aboriginal with risk factors (15–64 years)	2001–2007	196	(79.7)	3	(6.0)	47	(94.0)
Aboriginal (≥50 years)	2001–2007	16	(42.1)	9	(40.9)	13	(59.1)
Aboriginal with risk factors (15–49 years)	2001–2007	62	(49.2)	16	(25.0)	48	(75.0)

Note: Risk factors for children include immunodeficiency, chemotherapy or steroid use, renal failure, Down syndrome and preterm birth. Risk factors for adults include asplenia, immunodeficiency, chronic cardiac, renal or respiratory diseases and smoking [6].

^a^Proportions calculated from those with known vaccination status

^b^Funded immunisation programs with 7vPCV (for non-Aboriginal children) and 23vPPV (for non-Aboriginal adults aged 65 years or more) began in 2005 [7].

## 4. Discussion

Using retrospectively collected surveillance data, we described the clinical diagnosis, risk factors and comorbidities reported in Aboriginal and non-Aboriginal IPD cases from 1997 to 2007 throughout WA. Pneumonia was the most common clinical diagnosis in Aboriginal children and both Aboriginal and non-Aboriginal adult IPD cases in WA. A third of children and almost all adult cases of IPD had at least one risk factor or comorbidity. Congenital abnormality was the most common comorbidity in non-Aboriginal children while in Aboriginal children, preterm delivery was the most common risk factor. Cardiovascular disease and chronic respiratory disease were the most common comorbidities in non-Aboriginal adults while excessive alcohol consumption and smoking were the most common risk factors in Aboriginal adults with IPD.

National immunisation guidelines target groups who are at increased risk of contracting IPD [8]. In particular, adults with malignancy, chronic diseases, excessive alcohol use or who smoke are recommended to receive at least one dose of 23vPPV [8, 18]. Of those with known vaccination status, only 25% of Aboriginal and 6% of non-Aboriginal adults who were eligible for vaccination under these guidelines were vaccinated before contracting IPD. Vaccination status was unknown in a high proportion of adults, which makes it difficult to assess vaccine coverage and efficacy. It should be noted, however, that these findings are for people who contracted IPD and may therefore not be representative of vaccination coverage in the general WA population. Vaccine failures in this dataset have been previously documented [11].

Our previous analysis on this dataset has shown a higher incidence of IPD in Aboriginal adults aged 30–49 years than in similarly aged non-Aboriginal adults [11]. Here, we show that a higher proportion of Aboriginal adults with IPD smoked or consumed alcohol excessively compared to their non-Aboriginal counterparts or younger Aboriginal adults with IPD. These behaviours increase the risk of contracting IPD [19, 20] and also increase the risk of other chronic conditions, which can compound the risk of IPD [21]. Smoking, excessive alcohol use and associated conditions are markers of underlying issues in Aboriginal communities like dispossession, poverty, crowding and discrimination [22, 23]. Strategies to address these underlying issues as well as promoting vaccine uptake and smoking cessation in Aboriginal adults are needed.

Similar to a report from the United States [6], pneumonia was the most common clinical diagnosis among adults with IPD in this cohort. Interestingly, pneumonia was a more common clinical presentation among Aboriginal children with IPD than bacteraemia; elsewhere, the latter has been reported to be a more common clinical presentation for children [6]. These differences may be partially due to inconsistencies in criteria for collecting blood for culture (and therefore detection of IPD) in different parts of WA and around the world [24]. Although we have seen some reductions in hospitalisations for all-cause pneumonia in Aboriginal children, a significant disparity between Aboriginal and non-Aboriginal children for pneumonia remains in the WA population [25]. While the numbers of cases are small, we have reported here a high proportion of chronic respiratory disease among Aboriginal children with IPD, re-emphasising the increased risk of severe disease in children with recurrent respiratory infections [26].

While pneumonia has remained the most common clinical diagnosis in adults, the proportion of adult cases diagnosed with septicaemia has significantly increased over the two time periods, in particular for Aboriginal adults. The reasons for this are unclear but could be due to increased blood culture testing following the statutory notification of IPD cases that commenced in 2001. The proportion of Aboriginal and non-Aboriginal adults with cardiovascular disease, chronic respiratory disease and hypertension increased significantly in 2002–2007 compared to 1997–2001 (data not shown). In addition, the proportion of non-Aboriginal adults with malignancy as well as the proportion of Aboriginal adults who smoked or had diabetes increased significantly in 2002–2007 compared to earlier years (data not shown). These changes in risk profiles may reflect increased awareness of IPD, and hence improved documentation of risk factors following statutory notification and could have contributed to the observed increase in septicaemia diagnoses in 2002–2007.

Missing data, particularly missing vaccination data, is a limitation of this study. While this may be partly due to modification of the data collection sheet in 2001, reporting bias is also likely to have contributed to missing data. Data were collected from medical records of known IPD cases with no individual follow-up and were therefore, restricted to documented risk factors and comorbidities. Some factors such as chemotherapy or steroid use are more likely to be accurately reported in medical records than others (e.g. excessive alcohol use). Additionally, reporting of risk factors and comorbidities may be better in metropolitan regions than rural or remote regions, which may explain the observed higher proportions of adults with comorbidities in metropolitan regions. However, the proportions of cases with missing data on comorbidities were similar across geographical regions (data not shown). Future studies using population-based data linkage with a case-control study design may help to address issues with missing data [27].

We are unable to determine whether the proportion of IPD cases with risk factors and comorbidities reflected in the general population as there was no control group. As a general comparison, the proportion of adults in the general WA population who smoked (17%) or had chronic respiratory disease (approximately 2–3%) was lower than in adults with IPD [28]. This was similarly observed in the United States [19, 29]. Likewise, a greater proportion of Aboriginal adults with IPD in this study had diabetes or consumed alcohol at risky levels compared to the general Aboriginal population in Australia [30, 31], which is consistent with observations in Indigenous populations elsewhere [32]. This suggests that these risk factors and comorbidities are more common among adult IPD cases in WA than in the general population.

Conducted over a 10-year period, our study is the first to document risk factors and comorbidities in people with IPD in WA. A previous study on risk factors and comorbidities in Central Australia from 1980–2001 reported 74% of adults with IPD had one or more risk factors, including chronic diseases, petrol sniffing and alcohol abuse [20], which was lower than our findings. Similar studies in Latin America and Spain also found smoking and excessive alcohol consumption to be some of the most common risk factors for IPD [33, 34], adding to the validity of our study findings despite its limitations.

In July 2011, the 13-valent pneumococcal conjugate vaccine (13vPCV) replaced 7vPCV in the National Immunisation Program for children and 23vPPV as a booster for high risk children [18]. This study provides the baseline for evaluating changes in risk profiles in groups at high risk of IPD. Increased public health campaigns targeting known risk factors for IPD, such as the Break the Chain campaign targeting smoking [35], are needed, especially for the Aboriginal population. While it is important to continue to monitor the coverage and effectiveness of pneumococcal vaccination in children, there is still a need for a whole-of-life immunisation register for better assessment of vaccine coverage and effectiveness in preventing IPD in adolescents and in adults.
